# Analysis of Creep Properties and Factors Affecting Wood Plastic Composites

**DOI:** 10.3390/polym14142814

**Published:** 2022-07-10

**Authors:** Li Feng, Chunyan Zhao

**Affiliations:** College of Mechanical and Electrical Engineering, Northeast Forestry University, Harbin 150040, China; wang_lifeng100@nefu.edu.cn

**Keywords:** WPC, creep property, reliability, creep, load

## Abstract

Wood plastic composite (WPC) materials are mainly used as building slabs and load-bearing slabs, which will cause creep deformation, structural failure, and safety problems. Therefore, this work used high-density polyethylene and poplar wood flour as the main raw materials to prepare wood–plastic boards. The bending creep tests of wood–plastic sheets were carried out using an orthogonal test method. According to the creep test results, the influences of the WPC ratio molding temperature, pelleting temperature, coupling agent screw speed, and other technological factors on the creep properties of WPC composites under different loads are analyzed, and the influences of WPC creep properties on structural reliability are also analyzed. The results indicate that the wood–plastic ratio and screw speed are significant factors when the load is at 30% bending strength within the 24h creep test. When the load is at 50% bending strength, the wood–plastic ratio and molding temperature are the significant factors. When the load is at 70% bending strength, the wood–plastic ratio is the most significant factor. When the load is at 50% bending strength within the 240 h creep test, the wood–plastic ratio and molding temperature are significant factors. When the load is at 30% bending strength, the influence of each factor on the creeping variable is provided in the following descending order: wood–plastic ratio, molding temperature, granulation temperature, coupling agent, and screw speed, but none of them are significant factors.

## 1. Introduction

WPC is a kind of recyclable material that is mainly composed of biomass fiber, mixed with a certain proportion of plastic, and processed using a special process. It has the advantages of both wood and plastic. It is mainly used in construction, outdoor facilities, decorative materials, and other fields. It has become the ideal type of environmentally friendly material for residential and commercial purposes [1]. According to the moisture content measurement experiment, the moisture content of WPC is lower than that of wood, only 0.9%, which equates to the hydrophobicity of plastic. According to the ultimate volume expansion experiment, the ultimate volume expansion rate of WPC is 0.8%, which is much better than that of wood. According to the elastic modulus experiment, the elastic modulus of WPC is 4700 MPa, which is much higher than that of wood [2].

The WPC studied in this work is mainly used in the construction industry as load-bearing materials such as bridge plates. Due to the creep properties of WPC, the strain increases with time when the stress is constantly maintained. A large creep deformation will occur in the process of use, resulting in structural damage and safety problems. It is necessary for materials to have high mechanical properties and creep resistance. Therefore, in the process of material preparation, it is necessary to adjust the flexibility formulation and preparation technology, including the selection of the polymer matrix, the amount of wood fiber material, the selection of the coupling agent and extruder in temperature control, to reduce pressure and exhaust, to improve the screw shape, etc. It is also necessary to study the creep characteristics of WPC and clarify its creep law and influencing factors, and thus meet the demand of mechanical properties of WPC composites [3].

The effect of wood flour on the mechanical properties of eucalyptus/PVC composite has been studied by a creep test. The results indicate that when the particle size of the wood flour is 70–80 mesh, the mechanical properties of the composites are the best [4]. The effect of wood fiber on the creep resistance and mechanical properties of poplar/HDPE composites has been studied. The results reflect that when the size is 850–2000 μm, the long-term creep resistance of the composites is the best [5]. When increasing the wood flour content, the tensile strength of the composite increases then plateaus, and the impact strength and bending strength decrease [6,7,8]. The addition of wood flour improves the creep resistance and bending properties of the composites and decreases the creep deformation [9,10,11,12,13].

The tensile properties and impact strength of wood flour–polypropylene composite with or without a coupling agent has been investigated. The results show that the addition of a coupling agent increases the tensile properties and impacts strength [14]. By means of studying the creep resistance of WPC, the results indicate that the coupling agent can enhance the bending properties and creep resistance of the composites [15,16]. By studying the creep of wood flour–recycled polypropylene composites, the results reflect that the addition of a compatibilizer decreases the creep deflection and creep factor [17]. Studies of the short-term bending creep of polypropylene wood–plastic composites show that adding lubricating agents can greatly reduce the creep under high stress, and that the relative creep increases with an increasing temperature [18]. The effect of the impact modifier on the creep of wood flour–recycled polypropylene composites is studied through a short-term bending creep test. The results show that the impact modifier increases the creep deflection, the initial creep, the final creep, and the relative creep of composites containing the impact modifier and reduces the creep modulus [19].

Through studying the short-term creep of WPC under different load levels at room temperature, the results indicate that the creep variable is proportional to the load at room temperature and the creep curve is evidently non-linear. At the same time, the higher the load, the higher the creep rate [20,21,22,23]. At the beginning of the test, the stress level has little effect on the bending deformation growth rate of the WPC material. With an increasing stress level, the strain growth rate of the material gradually increases. The creep variable of WPC is relatively small when the load stress is relatively small (<30%). When the load stress is more than 30% bending strength, the creep variable increases significantly and even breaks within a short time [24]. By studying the creep of a bagasse-based composite with untreated and recycled polyvinyl chloride (B/PVC) and High-Density Polyethylene (B/HDPE), as well as commercial wood and HDPE composite decking materials, the results show that the instantaneous deformation and creep rate of all composites increase at higher temperatures with the same load level. At a constant load level, B/PVC composite has better creep resistance at low temperatures than B/HDPE systems [25].

Creeping occurs when loads are applied for a long period of time and leads to the fracture of the WPC board structure. Therefore, to maintain lasting competitiveness and expand the scope of its use, WPC must first meet the requirements of reliability and durability. In this work, in order to improve the creep resistance of WPC under a constant room temperature, changing the wood–plastic ratio, coupling agent, forming factors (such as temperature), and screw speed, reveals the factors affecting the creep property of wood–plastics composites, and analyzes the creep under different load effects on the reliability of the wood–plastic composite.

## 2. Materials and Methods

In this work, polyethylene and poplar powder were used as main raw materials to prepare wood–plastic specimens. The wood flour was produced by Qingdao Fujilin New Energy Co., Ltd. (Qingdao, China), with a particle size of 80 mesh. The PE was produced by Daqing Petrochemical, with a density of 0.954 g/cm^3^ and a melt index of 0.9 g/10 min. The PE was supplied in the form of homopolymer pellets. The coupling agent was CMG9804, which was a graft HDPE compatibilizer produced by Nantong Ri Zhi Sheng Polymer New Material Technology Co., Ltd. (Nantong, China).

In this work, the melt extrusion method was used to prepare test specimens. The high-speed mixer was SRH-10A, produced by Zhangjiagang Tongsha Plastic Machinery Factory (Zhangjiagang, China). The twin-screw mixing extruder was the SJSH-30, produced by Nanjing Rubber and Plastic Machinery Factory (Nanjing, China). The single-screw extruder was SJ-45, produced by Nanjing Rubber and Plastic Machinery Factory (Nanjing, China). 

In this work, molding temperature, screw speed, WPC mass ratio, coupling agent content, and granulation temperature were taken as process factors, and orthogonal test method was adopted to design the preparation process factors of the WPC specimen, as shown in Table 1. The corresponding ratios of wood flour and PE were 55:45, 60:40, 65:35, and 70:30. The coupling agent contents were 2%, 3%, 4%, and 5% base on the total weight of wood flour and PE. During specimen preparation, the granulation temperature and molding temperatures were 150 °C, 160 °C, 170 °C, and 180 °C. The screw speeds were 30, 50, 70, and 90 rpm.

**Figure 1:**
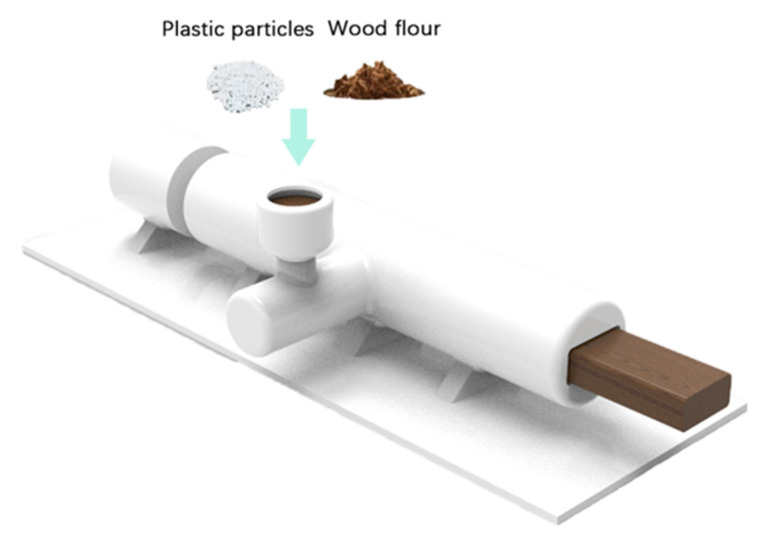
Process diagram of extrusion molding method.

The wood flour was placed in an electric thermostatic drying oven at 80 °C for 12 h until the water content was less than 2%. After the drying of wood flour, the weights of the wood flour, polyethylene, and the coupling agent after grinding were measured according to Table 1. Poplar wood flour, polyethylene, and coupling agent were mixed in a high-speed mixer for 10min and then poured into a twin screw extruder for granulation. Different granulation temperatures and screw rotation speeds were adjusted according to Table 1. After granulation, the material was poured into a single screw extruder to extrude the WPC specimen, as shown in Figure 1.

According to ASTM D790 standard [26], the specimens were cut into boards with dimensions of 80 mm × 13 mm × 4 mm. The bending load was recorded by means of three-point bending load test, and the bending strength of each group of specimens was calculated. The results are shown in Table 1.

## 3. Creep Test

**Figure 2:**
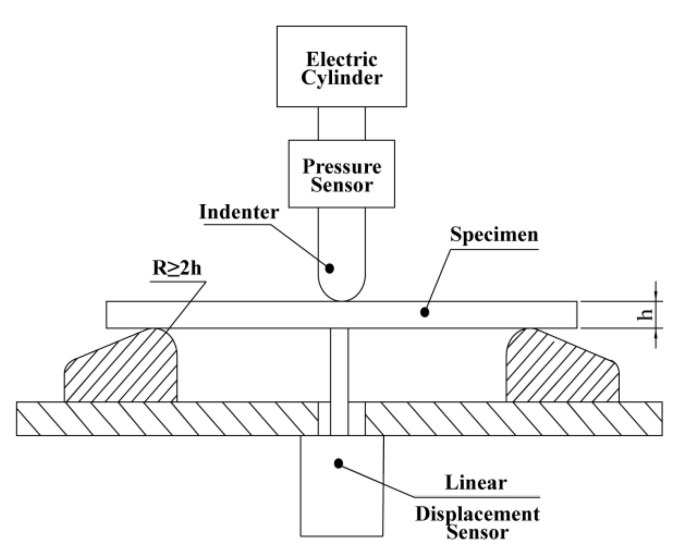
Schematic diagram of creep test.

The creep test in this work referred to the ASTM790 standard. The creep test device adopted a vertical bench, where the upper part was the loading device, and the lower part was the data acquisition device [27]. Since the maximum bending strength of the wood–plastic composite material was 63.97 MPa, far lower than the traditional metal material, the design of the test bench mainly considered the structural problem of ensuring the realization of the bench function, while the strength and stiffness of the bench were secondary considerations. The schematic diagram of the creep test device is shown in Figure 2.

The short-term 24 h bending creep test refers to the ASTM D7031 [28] standard (creep recovery and creep failure). The number of test specimens in each group was not less than 10, and each specimen was loaded for 24 h and then unloaded for another 24 h. The bending deflection of the midspan was measured before loading, after loading for 24 h, after unloading for 1 min, and after recovery for 24 h. The number of test specimens in each group was five. The creep time of each specimen was set at 10 days. The loads were 30%, 50%, and 70% of the bending strength of the tested specimen. Other parameters such as specimen size, span, etc., referred to the ASTM D790 Standard.

## 4. Results and Discussion

The average strain of the five specimens was taken as the final creep variable and the specimens were investigated for the presence of fracture phenomena. Due to the large amount of data, it is impossible to list them all; therefore, the partial data are shown in Table 2.

### 4.1. Influencing Factors of 24 h Creep of Wood–Plastic Board

**Figure 3:**
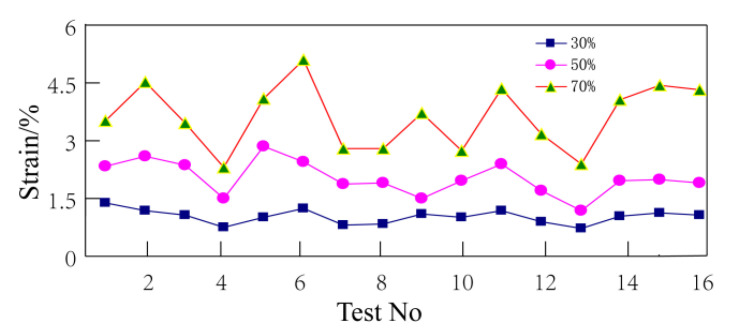
Final creep-strain after 24 h: The loads are 30%, 50%, and 70% bending strength of wood–plastic boards.

The loads were 30%, 50%, and 70% bending strength of wood–plastic boards. Since all specimens broke within 24 h when the load was 70% bending strength, the strain before fracture was taken as the final strain. The final strain after 24 h creep test is shown in Figure 3.

As shown in Figure 3, the strain curves have quite similar trends when the load is at 30%, 50%, and 70% bending strength. However, the strains vary considerably, and even increase several times under different loads. The load is one of the important factors affecting the creep variable of wood–plastic boards.

To accurately analyze the influence of the various process factors on the creep variable of the wood–plastic boards within 24 h, a regression analysis was carried out on the 24 h strain variables and process factors when the loads were 30%, 50%, and 70% bending strength. The results are shown in Table 3, Table 4 and Table 5. The significant process factors are selected and the influence rule of each significant process factor on 24 h creep is analyzed.

It can be observed from Table 3 that the absolute values of the standardized coefficients are 0.154, 0.357, 0.771, 0.050, and 0.215. Therefore, when the load is at 30% bending strength, the degree of influence of each process factor on the 24 h creep variable is provided in descending order as follows: wood–plastic ratio, screw speed, granulation temperature, molding temperature, and coupling agent. The Sig value of the wood–plastic ratio and the screw speed is less than 0.05, indicating that the influence on the 24 h creep with the load of 30% bending strength is significant. While the influence of other process factors on the dynamic elastic modulus is relatively small, from the significance point of view, the significance of the wood–plastic ratio is much greater than the screw speed.

It can be observed from Table 4 that the absolute values of the standardized coefficients are 0.436, 0.191, 0.602, 0.023, and 0.092. Therefore, when the load is at 50% bending strength, the degree of influence of each process factor on the 24 h creep variable is noted in descending order as follows: wood–plastic ratio, molding temperature, screw speed, granulation temperature, and coupling agent. In the process factors, the wood–plastic ratio and the molding temperature Sig. values are less than 0.05, so the influence on the 24 h creep variable with the load of 50% bending strength is significant, while the influence of other process factors on the dynamic elastic modulus is relatively small.

An examination of the data presented in Table 5 shows that the absolute value of the standardized coefficients is 0.115, 0.158, 0.818, 0.222, and 0.092. Therefore, when the load is at 70% bending strength, the degree of influence of various process factors on the 24 h creep variable is given in descending order as: wood–plastic ratio, coupling agent, screw speed, molding temperature, and granulation temperature. In the process factor, the Sig. value of the wood–plastic ratio is less than 0.05, so the influence on the 24 h creep variable with the load of 70% bending strength is extremely significant, while the influence of other process factors on the dynamic elastic modulus is relatively small.

With the above analysis, it is evident that under different loading conditions the influence of various process factors on creep is different. When the load is at 30% bending strength, the wood–plastic ratio and screw speed are significant factors. However, when the load is at 50% and 70% bending strength, although each process factor has a different significance on creep variable, one common feature is that only one significant factor affects the creep (the wood–plastic ratio). The wood–plastic ratio is the most significant influencing factor under these three loads, and an examination of the data presented in Figure 3 shows that the corresponding test numbers of plates with small strain are 4, 7, 8, 10, and 13. Observing the orthogonal test table, it is evident that the formulation of these samples have a remarkable feature: the wood flour content is relatively high, especially the wood flour contents of No. 4, 7, 10, and 13, which are up to 70%. Therefore, the composition properties of the material itself plays a decisive role in the creep properties of wood–plastic boards. The reasons are as follows: HDPE is a linear polymer with low intermolecular force and good flexibility. The addition of wood powder particles increases the intermolecular resistance of HDPE. Under the action of an external force, wood powder can withstand greater stress, and uses its characteristics and matrix to transfer stress, which plays a strengthening role.

### 4.2. Analysis of Influencing Factors of 240 h Creep Variable of Wood–Plastic Board

**Figure 4:**
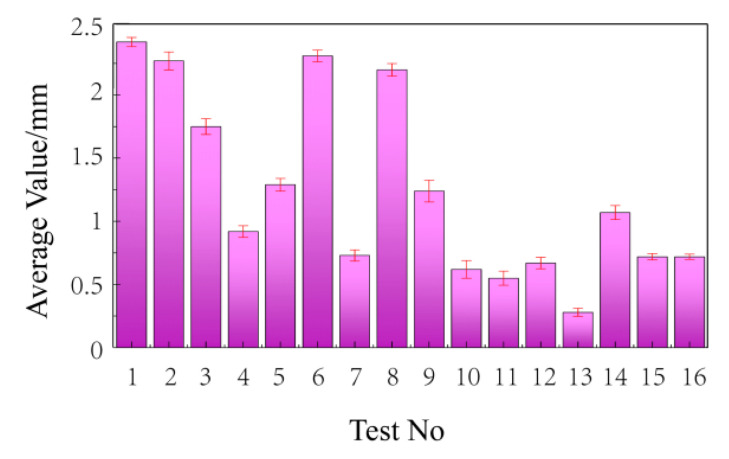
Creep variable at 240 h.

Since the creep of 24 h to 240 h is relatively small, the expression of the strain percentage is not conducive to observation. To more intuitively express the deformation of wood–plastic boards, the deformation is characterized by the deflection change of the wood–plastic boards. The creep variable of the fracture specimens at 240 h with the average strain before fracture is shown in Figure 4.

The creep variables as shown in Figure 4 are analyzed by means of a regression analysis. The regression coefficient is shown in Table 6 to clarify the influence of different process factors on the 240-h creep of the wood–plastic boards. The absolute values of the standardization coefficient are 0.686, 0.187, 0.392, 0.001, and 0.253. When the load is at 50% bending strength, the influence of each process factor on the 240-h creep variable in descending order is: molding temperature, wood–plastic ratio, granulation temperature, screw speed, and coupling agent. Among the process factors, the Sig. values of the molding temperature and the wood–plastic ratio are less than 0.05. The influence on the 240-h creep variable with a load of 50% bending strength is significant, while the influence of other process factors on the dynamic elastic modulus is relatively small.

### 4.3. Analysis of Creep Law

With the analysis of the 24 h creep variable of wood–plastic boards, it was found that the wood–plastic ratio and screw speed are the significant factors, except for the 30% bending strength under load. The wood–plastic ratio is the most significant influencing factor on the 24 h creep under the other two stresses. Therefore, the wood–plastic ratio is the primary technological factor affecting the 24 h creep of wood–plastic boards, so it is necessary to analyze the influence rule of the wood–plastic ratio on the creep of wood–plastic boards. As wood–plastic composite plate is a viscoelastic material with elastic deformation, its viscous deformation and viscoelastic deformation are different from general solid materials. To clarify the deformation law of each part, the deformation curves of wood–plastics plate under different loads and at different times are analyzed.

#### 4.3.1. Effect of Wood–Plastic Ratio on 24 h Creep Variable

**Figure 5:**
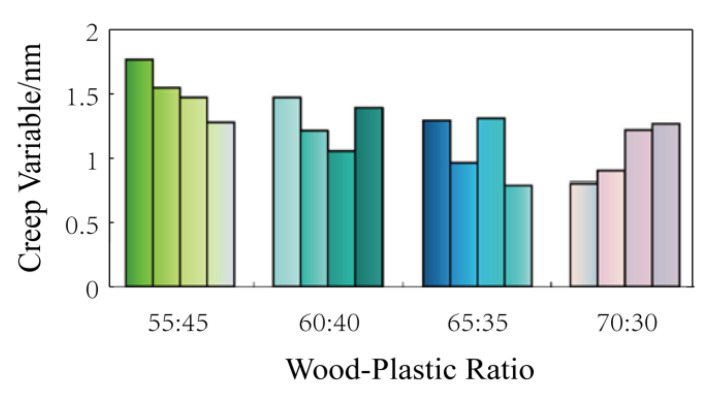
Relationship between wood–plastic ratio and creep: There are four schemes in one group, and wood–plastic ratio of each group is the same.

According to the regression analysis results of the technological factors and 24 h creep variable, the wood–plastic ratio is the most important factor affecting the 24 h creep variable. It is evident from Figure 3 that the trends of the 24 h creep variable under the three loads have similar characteristics. With the curves of 30% and 70% bending strength, it is evident that the specimens with a smaller strain are No. 4, 7, 10, and 13, while those with 50% bending strength are No. 4, 7, 9, and 13. By comparing the test factors in the orthogonal test table, it is evident that there is a common feature wherein the wood–plastic ratio is larger (i.e., the wood flour content is higher). The 16 kinds of boards were divided into four groups according to the requirement for the ratio of wood to plastic, and the creep strain variables were observed. The first group is Formula No. 1, 6, 11, and 16 (wood–plastic ratio 55:45); the second group is Formula No. 2, 5, 12, and 15 (wood–plastic ratio 60:40); the third group is Formula No. 3, 8, 9, and 14 (wood–plastic ratio 65:35); and the fourth group is Formula No. 4, 7, 10, and 13 (wood–plastic ratio 70:30). Since the deformation tendencies of each load are similar, the creep data with a load of 30% bending strength was selected for observation as presented in Figure 5.

In Figure 5, the wood–plastic ratio of each group is the same, and the creep variables of the four schemes in each group are relatively close, while there is a downward trend between groups. An examination of the data presented in Figure 5 shows that the creep of the wood–plastic boards decreases with the increase of wood–plastic ratio. That is, the higher the wood flour, the content in the range of 55:45–70:30 wood–plastic ratio, the lower the creep deformation of the material. The same conclusion is also reflected in this work where increasing the content of wood fiber in a certain temperature range is beneficial to inhibit the creep strain of WPC [29]. 

It is also evident from Figure 5 that with the increase of the wood–plastic ratio, the influences on their creep variables also gradually decrease. When the wood–plastic ratio is greater than (60:40), the influences on the 24 h creep variable slow down with the increase of the wood flour, a tendency that is even less obvious in some cases.

#### 4.3.2. Effect of Wood–Plastic Ratio on 24 h Creep Trend

**Figure 6:**
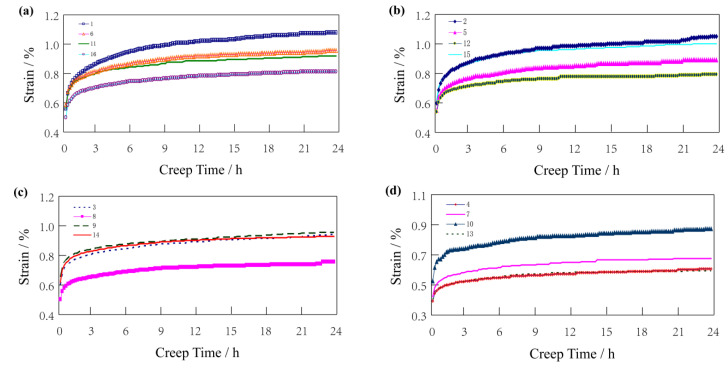
Relationship between wood–plastic ratio and 24 h creep variable: (**a**) relationship between wood–plastic ratio (55:45) and 24 h creep variable; (**b**) relationship between wood–plastic ratio (60:40) and 24 h creep variable; (**c**) relationship between wood–plastic ratio (65:35) and 24 h creep variable; (**d**) relationship between wood–plastic ratio (70:30) and 24 h creep variable.

Using the above analysis, the wood–plastic ratio is the most significant factor affecting the 24 h creep variable of the wood–plastic board. According to the wood–plastic ratio, the 24 h creep variables of the wood–plastic board were divided into four groups. The influence of the wood–plastic ratio on the creep trend of the wood–plastic board for 24 h was analyzed. Since the graphs of the creep strain under various loads are similar, the creep strain cases with a load of 30% bending strength are listed, as shown in Figure 6.

It can be observed from Figure 6 that when the applied load is at 30% bending strength, the 24 h creep trends of the various formulations are quite similar and show three stages. The first stage is from the beginning of loading to about 60 min after loading. The curve corresponding to this stage has the largest curvature. The second stage is from 60 min to about 600 min. The curvature of the deformation curve in this stage gradually becomes smaller, which shows a decreasing strain rate. In the process of reduction, the third stage is from 600 min to the end of the experiment, and the creep speed tends to a relatively stable state. The duration of each stage varies with the wood–plastic ratio.

The first stage: with the increase of the wood–plastic ratio, the curvature of the deformation curve of the first stage is smaller, especially the creep curve of the No. 13 specimen in Figure 6d. With the increase of the wood–plastic ratio, the strain corresponding to the first stage gradually reduces, and the second stage will involve a shorter time.

The second stage: with the increase of the wood–plastic ratio, the amount of strain time and strain in the second stage decreases. When the wood–plastic ratio is larger, it tends to reach the third stage of steady state faster.

The third stage: when the specimens enter the third stage, the curvature of the deformation curve shows a relatively stable state. However, with the increase of the wood–plastic ratio, the strain curve corresponding to this stage is straighter and the strain variable is smaller. In the 24 h creep test with the load of a 30% bending strength, the screw speed is the second significant factor in the formula with a small wood–plastic ratio. Therefore, it is necessary to analyze the effect of screw speed on the creep variable.

From Figure 6a, it can be seen that the corresponding strains under the same wood–plastic ratio are divided into different heights, and the influence of screw speed on creep is investigated in the corresponding factor level table. It is evident that the creep of the boards with these four formulations decreased with the increase in the screw speed for 24 h and it is quite consistent. This can be explained by the fact that under the formula of the wood–plastic ratio, with the increase of the screw speed, the extrusion pressure increases greatly, resulting in the gradual decrease of the internal gap of the extruded plate, and the internal structure becomes dense and stable. By comparing the density corresponding to the four formulas, it is found that when the screw speed is relatively high, it has a greater density. However, with the increase in the wood–plastic ratio, this characteristic gradually becomes less obvious. When the wood–plastic ratio is (60:40) and (65:35), the large screw speed (90 rpm) has a relatively small creep variable. At this time, the influence of the screw speed (30, 50, and 70 rpm) on the creep variable is not significant. When the wood–plastic ratio is (70:30), the effect of the screw speed on the creep variable is relatively small. It can be seen from the information above that when the wood–plastic ratio is small, the screw speed plays an obvious role in reducing the internal pores of the wood–plastic plate. However, with the increase of the wood–plastic ratio, the extrusion becomes more difficult, and the internal extrusion pressure reaches a relatively high level. At this time, the effect of a lower screw speed on improving the internal structure of the wood–plastic plate is not obvious, and only a higher screw speed can reflect this effect. When the wood–plastic ratio reaches (70:30), the screw speed has little effect on improving the internal structure of wood–plastic plate.

### 4.4. Analysis of 24 h Creep Recovery Trend

**Figure 7:**
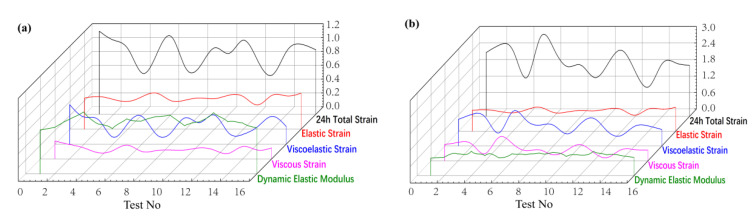
Creep recovery parameters for 24 h: (**a**) load: 30% bending strength; (**b**) load: 50% bending strength. The residual strain after 24 h of creep recovery is regarded as viscous strain. Using Hooke’s law, the remaining strain is considered as viscoelastic strain. The 24 h total strain is the sum of elastic strain, viscoelastic strain, and viscous strain.

In 24 h creep deformation under the loads of 30% and 50% bending strength, 24 h recovery tests were carried out on the wood–plastic board. After the load is removed, the deformation of the elastic solid can return to its initial state. However, the viscous body has no possibility of deformation recovery. The stress of the elastic solid is directly related to the strain, while the stress in viscous fluid is related to the strain rate. WPC is a kind of viscoelastic body, which has elastic deformation, viscous deformation, and viscoelastic deformation. In this test, the residual strain after 24 h of creep recovery is regarded as viscous strain. The elastic deformation part is assumed to be an ideal elastomer, which is calculated by Hooke’s law, and the remaining strain is regarded as viscoelastic strain. Since the creep of wood–plastic composite has a viscoelastic deformation behavior and the elastic modulus is the scale to measure the deformation resistance of materials, it is necessary to take the elastic modulus as a reference factor for the creep deformation of wood–plastic composite. Due to the high correlation between the dynamic elastic modulus and elastic modulus, it can replace the elastic modulus. The test results are shown in Figure 7.

From Figure 7, it is evident that the 24 h total strain of the specimens with a higher dynamic elastic modulus is smaller. The elastic strain of the specimens with a higher dynamic elastic modulus account for a larger proportion of the 24 h total strain. Therefore, increasing the dynamic elastic modulus of WPC is beneficial to the occurrence of less creep and the stability of the structure. From the test results, it is evident that the elastic strain, viscous strain, and viscoelastic strain of WPC are highly correlated with the composition of the material itself. Therefore, the dynamic elastic modulus is an important reference factor for creep deformation of WPC.

**Figure 8:**
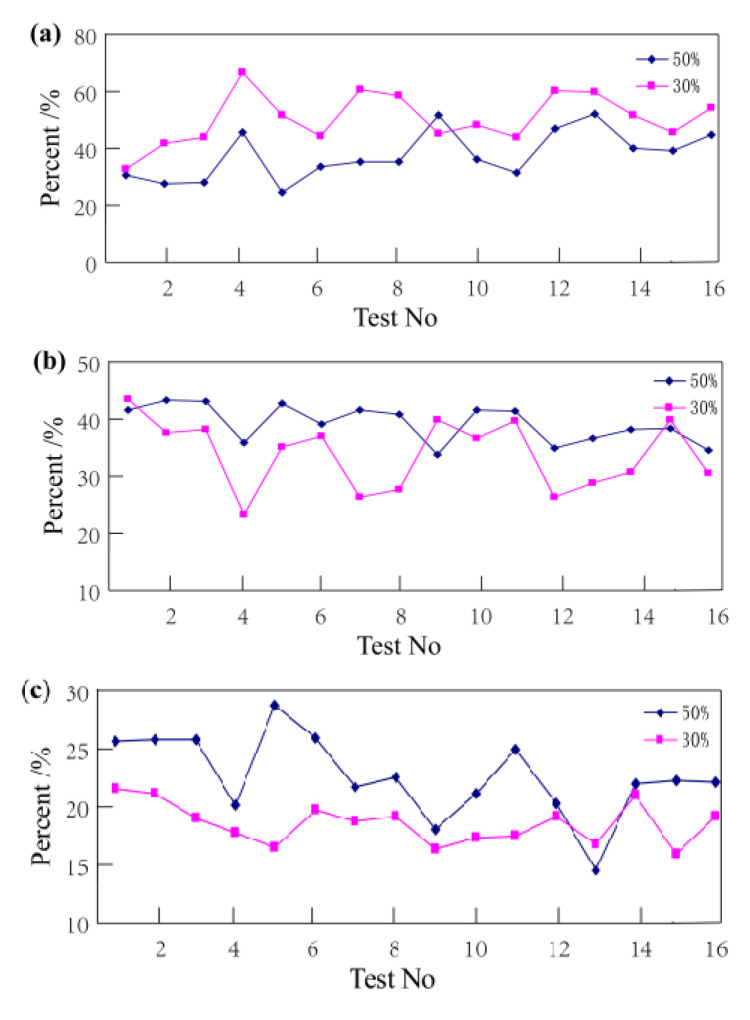
Various strain ratios of WPC for 24 h: (**a**) the proportion of elastic deformation; (**b**) the proportion of viscoelastic deformation; (**c**) the proportion of viscous deformation.

The wood–plastic ratio has similar properties for the creep deformation of wood–plastic composite. With the increase of the wood flour, the elastic deformation of the plates and the recovery rate of the creep increases, and the relative viscous deformation decreases. With respect to increasing the load, the proportion of elastic strain, viscoelastic strain, and viscous strain relative to the total strain of WPC is shown in Figure 8.

Figure 8a shows that when the load is at 30% bending strength, the elastic deformation of all 16 formulations is the largest among the three types of deformation and the elastic deformation of the 8 formulations is greater than 50%. It also shows similar characteristics when the load is at 50% bending strength, which indicates that the elastic deformation is the most important deformation in 24 h creep deformation of WPC. It can also be seen from Figure 8b that with the increase of the load, the proportion of elastic strain to the total strain decreases, which is also be reflected by Figure 8b,c. The viscoelastic and viscous deformation of WPC increase with the increase of load. According to the above three figures, it was found that the elastic strain > viscoelastic strain > viscous strain in the 24 h creep deformation of the wood–plastic board.

### 4.5. Influence of Creep of Wood–Plastic Composites on Structural Reliability

Since the creep characteristics of WPC will affect the material’s reliability, the first order second moment method is used to calculate the material reliability of wood–plastic boards. The structural reliability indexes and reliability calculation results under different formulas and different loads are shown in Table 7.

After calculation, the reliability of all wood–plastic panels with the load of 30% bending strength is one, and no boards are damaged by creep during the 240-h creep test, indicating that the reliability of wood–plastic panels with the above formula is 100% with the load of 30% for 240 h. With the load of 70% bending strength, all the plates will break within 10 h, so the creep reliability is zero for 240 h. The 240-h creep reliability with the load of 30% bending strength is 100%. The 240-h creep reliability with the load of 70% bending strength is zero. Therefore, the influence of creep on the reliability of WPC under the load of 50% bending strength was emphasized. The fracture of each formula with the load of 50% bending strength is shown in Table 8.

**Figure 9:**
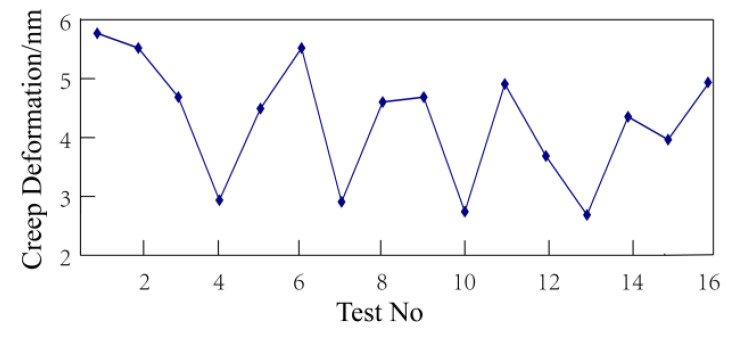
Creep with load (50% bending strength) for 240 h.

From the data in Table 8, we can see the fracture situations of various formula sheets, among which No. 3, 4, 5, 7, 10, and 13 sheets all have fracture behaviors in the 240-h creep test. By observing the factor level table, it can be found that the wood–plastic ratio of Formula 4, 7, 10, and 13 is larger (70:30). From the above results, it can be seen that only the formula with the wood–plastic ratio (55:45) does not break. In addition, from the test process, it is shown that the highest fracture rate is the formula with the wood–plastic ratio (70:30). Figure 9 shows the final deformation of the creep test with the load of 50% bending strength after 240 h. For fracture specimens, the average data before fracture is taken as the final data.

It can be seen from Figure 9 that the creep variables of No. 4, 7, 10, and 13 formulations with the highest fracture ratio are relatively small. The creep variables of No. 1, 6, 11, and 16 formulations without fractures are the largest. The final creep variables of the remaining formulations are similar. Therefore, within a certain wood–plastic ratio range (55:45–70:30), the larger the wood–plastic ratio, the smaller the creep variable and the lower the material’s reliability. The same conclusion is also reflected in the cited work, where the wood powder content is higher than 60%, and the reliability decreases with the increase of wood powder content [30].

## 5. Conclusions

In this work, the influence of process factors such as wood–plastic ratio, molding temperature, granulation temperature, the coupling agent, and screw speed on the creep of WPC and the influence of creep of WPC on the structural reliability were analyzed by means of a multiple regression method when the load was at 30%, 50%, and 70% bending strength. The results show that:

(1) The effects of various technological factors on the creep properties of WPC are different under different loading conditions, but the wood–plastic ratio is the most significant factor. In the range of (55:45–70:30), the higher the wood powder content, the lower the creep of the material. When the wood–plastic ratio is greater than (60:40), the influence of wood powder on the 24 h creep slows down or even becomes insignificant.
(2) In the 24 h creep deformation of WPC, the elastic deformation, viscoelastic deformation, and viscous deformation of WPC increase with the increase of the load, and the most important deformation is the elastic deformation, and the proportion of the elastic strain in the total strain decreases gradually with the increase of load.
(3) When the load is small (30% bending strength), the wood–plastic sheet creep reliability is the highest. When the load increases, the reliability begins to reduce. Some WPC boards began to fracture during the creep test at the load of 50% bending strength. All wood–plastic panels show creep fracture within a short time when the load is at 70% bending strength, and the higher the wood–plastic ratio is, the lower the creep reliability is and the easier it is to fracture.

This work solely considers the influence of creep characteristics of WPC on reliability at room temperature and constant load. In practical applications, environmental factors such as humidity, temperature, sunlight exposure, and alternating loads will also have an impact. To study the reliability of WPC more effectively, it is necessary to continue to study the prediction methods of the structural reliability of WPC and accurately calculate the influence of creep on the reliability through a large number of test data in future research.

## Figures and Tables

**Table 1:** Orthogonal test table for specimen preparation.

Test No	Molding Temp/°C	Screw Speed r/min	Wood–Plastic Ratio	Coupling Agent/%	Granulation Temp/°C	Bending Strength/MPa
1	150	30	55:45	2	150	55.19
2	150	50	60:40	3	160	58.25
3	150	70	65:35	4	170	58.95
4	150	90	70:30	5	180	63.14
5	160	30	60:40	4	180	58.05
6	160	50	55:45	5	170	58.45
7	160	70	70:30	2	160	59.55
8	160	90	65:35	3	150	62.06
9	170	30	65:35	5	160	63.97
10	170	50	70:30	4	150	62.45
11	170	70	55:45	3	180	58.43
12	170	90	60:40	2	170	53.47
13	180	30	70:30	3	170	51.35
14	180	50	65:35	2	180	60.14
15	180	70	60:40	5	150	60.16
16	180	90	55:45	4	160	57.33

**Table 2:** Creep data.

Time	Strain 1	Strain 2	Strain 3	Strain 4	Strain 5	Strain 6	Strain 7	Strain 8
1 min	0.59	0.53	0.50	0.35	0.47	0.62	0.39	0.38
10 min	0.72	0.63	0.60	0.41	0.57	0.74	0.45	0.45
20 min	0.78	0.69	0.64	0.43	0.60	0.78	0.47	0.47
⋮	⋮	⋮	⋮	⋮	⋮	⋮	⋮	⋮
1420 min	1.28	1.07	0.94	0.59	0.88	1.12	0.66	0.70
1430 min	1.28	1.07	0.94	0.59	0.88	1.12	0.66	0.70
1440 min	1.28	1.07	0.94	0.59	0.88	1.12	0.66	0.70

**Table 3:** Regression coefficient (load:30% bending strength).

Model	Unstandardized Coefficients		Standard Coefficient	t	Sig.
	B	Standard Error			
(Constant)	4.623	1.017		4.547	0.001
Molding Temp	−0.005	0.004	−0.154	−1.078	0.306
Screw Speed	−0.005	0.002	−0.357	−2.493	0.032
Wood–plastic Ratio	−0.607	0.113	−0.771	−5.381	0.000
Coupling Agent	0.015	0.042	0.050	0.347	0.736
Granulation Temp	−0.006	0.004	−0.215	−1.502	0.164

**Table 4:** Regression coefficient (load:50% bending strength).

Model	Unstandardized Coefficients		Standard Coefficient	t	Sig.
	B	Standard Error			
(Constant)	9.925	3.481		2.851	0.017
Molding Temp	−0.031	0.014	−0.436	−2.172	0.055
Screw Speed	−0.007	0.007	−0.191	−0.949	0.365
Wood–plastic Ratio	−1.158	0.386	−0.602	−2.996	0.013
Coupling Agent	−0.016	0.143	−0.023	−0.113	0.912
Granulation Temp	0.007	0.014	0.092	0.455	0.658

**Table 5:** Regression coefficient (load:70% bending strength).

Model	Unstandardized Coefficients		Standard Coefficient	t	Sig.
	B	Standard Error			
(Constant)	3.784	2.971		2.741	0.232
Molding Temp	0.009	0.12	0.115	0.751	0.470
Screw Speed	−0.006	0.006	−0.158	−1.029	0.328
Wood–plastic Ratio	−1.762	0.330	−0.818	−5.334	0.000
Coupling Agent	0.177	0.122	0.222	1.445	0.179
Granulation Temp	0.007	0.012	0.092	0.603	0.560

**Table 6:** Regression coefficient (240 h, load:50% bending strength).

Model	Unstandardized Coefficients		Standard Coefficient	t	Sig.
	B	Standard Error			
(Constant)	12.423	2.522		4.925	0.001
Molding Temp	−0.043	0.01	−0.686	−4.127	0.002
Screw Speed	−0.006	0.005	−0.187	−1.126	0.287
Wood–Plastic Ratio	−0.661	0.28	−0.392	−2.359	0.04
Coupling Agent	0	0.104	−0.001	−0.005	0.996
Granulation Temp	−0.016	0.01	−0.253	−1.525	0.158

**Table 7:** Reliability index (β) and reliability ($P_{r}$).

Test No	1	2	3	4	5	6	7	8	9	10	11	12	13	14	15	16
β 50%	17.9	19.3	17.7	10.3	15.7	19.8	15.7	18.1	19.2	19.7	19.9	17.2	17.6	17.6	17.3	19.3
$P_{r}$ 50%	1	1	1	1	1	1	1	1	1	1	1	1	1	1	1	1
β 70%	6.25	8.41	8.01	5.52	7.45	8.52	7.45	8.11	8.39	8.5	8.55	7.89	7.98	7.99	7.9	8.4
$P_{r}$ 70%	1	1	1	0.99	1	1	1	1	1	1	1	1	1	1	1	1

**Table 8:** Fracture condition.

1	2	3	4	5	6	7	8	9	10	11	13	14	15	16
-	-	fracture	fracture	fracture	-	fracture	-	-	fracture	-	fracture	-	-	-

## Data Availability

Not applicable.

## References

[1] Tang J., Lu Q., Yuan J. (2019). Study on the properties of PP bamboo plastic composites toughened by white carbon black and mPE. J. Mater. Sci. Eng..

[2] Chen X.X. (2013). Study on the Application of Plastic Wood Composite Materials in Furniture.

[3] Sun H., Lv X., Yuan N., Wang Q., Hao X., Sun L. (2021). Strength analysis and extrusion process optimization of wood-plastic composite by response surface method. J. Compos. Mater..

[4] Song L.X., Zhang P., Yao N.N., Song Y.Z., Kang M., Song K.P. (2013). Study on the influence of particle size and filling amount of wood powder on the mechanical properties of wood-plastic composites. Funct. Mater..

[5] Xu H.L., Cao Y., Wang W.H., Wang Q.W., Wang H.G. (2016). Effect of poplar fiber size on mechanical and creep properties of hot pressed poplar fiber/HDPE composites. J. Compos. Mater..

[6] Hao X., Yi X., Sun L., Wang Q., Ou R. (2019). Mechanical properties, creep resistance, and dimensional stability of core/shell structured wood flour/polyethylene composites with highly filled core layer. Constr. Build. Mater..

[7] Homkhiew C., Ratanawilai T., Thongruang W. (2012). Effect of Wood Flour Content and Cooling Rate on Properties of Rubberwood Flour/Recycled Polypropylene Composites. Adv. Mater. Res..

[8] Avu V., Mengeloglu F. (2020). Effect of Wood Particle Size on Selected Properties of Neat and Recycled Wood Polypropylene Composites. Bioresources.

[9] Xue J., Xue P. (2010). Study on creep resistance of HDPE/wood flour composites. Appl. Eng. Plast..

[10] Lee S.Y., Yang H.S., Kim H.J., Jeong C.S., Lim B.S., Lee J.N. (2004). Creep behavior and manufacturing parameters of wood flour filled polypropylene composites. Compos. Struct..

[11] Bledzki A.K., Faruk O. (2004). Creep and impact properties of wood fibre–polypropylene composites: Influence of temperature and moisture conten. Compos. Sci. Technol..

[12] Mosiewicki M.A., Marcovich N.E., Aranguren M.I. (2011). Creep behavior of wood flour composites made from linseed oil-based polyester thermosets. J. Appl. Polym. Sci..

[13] Jia M., Xue P., Zhao Y., Zhang K. (2009). Creep Behaviour of Wood Flour/Poly(vinyl chloride) Composites. J. Wuhan Univ. Technol. Mater. Sci. Ed..

[14] Febrianto F., Hidayat W., Wistara I., Park S., Kim N. (2017). Influence of Impact Modifier–Coupling agent Combination on Mechanical Properties of Wood Flour–Reinforced Polypropylene Composit. J. Fac. Agric. Kyushu Univ..

[15] Wang K.J., Zhao Y.S., Zhu F.H. (2007). Flexural and creep properties of wood plastic composites filled with montmorillonite. Polym. Mater. Sci. Eng..

[16] Huang C.W., Yang T.C., Wu T.L., Hung K.C., Wu J.H. (2018). Effects of maleated polypropylene content on the extended creep behavior of wood-polypropylene composites using the stepped isothermal method and the stepped isostress method. Wood Sci. Technol..

[17] Saman G., Saeed K. (2013). A Study on Creep Behavior of Wood Flour- Recycled Polypropylene Composite. Iran. J. Wood Pap. Ind..

[18] Park B.D., Balatinecz J.J. (2010). Short term flexural creep behavior of wood-fiber/polypropylene composites. Polym. Compos..

[19] Saman G., Behbood M., Saeed K.N. (2016). Effect of Ethylene Vinyl Acetate as an Impact Modifier on Creep Behavior of Wood Flour- Recycled Polypropylene Composites. J. For. Wood Prod..

[20] Homkhiew C., Ratanawilai T., Thongruang W. (2014). Time–temperature and stress dependent behaviors of composites made from recycled polypropylene and rubberwood flour. Constr. Build. Mater..

[21] Jiang Y.T., Li K., Wu Z.Y., Ding J.S. (2008). Study on creep properties of rice husk/HDPE wood plastic composites. Packag. Eng..

[22] Dong Z.X. (2010). Simulation and prediction of creep behavior of polypropylene wood plastic composite. Polym. Mater. Sci. Eng..

[23] Xu H.L., Cao Y., Li L.F. (2019). Creep properties of Masson Pine and Chinese fir reinforced polymer composites. J. Fujian For. Univ..

[24] Tian X.L., Li K., Jiang Y.T., He Q., Wu C.Y. (2008). Creep behavior of wood plastic composites under different loading modes. Plast. Ind..

[25] Xu Y., Wu Q., Yong L., Fei Y. (2010). Creep behavior of bagasse fiber reinforced polymer composites. Bioresour. Technol..

[26] (2010). Standard Test Methods for Flexural Properties of Unreinforced and Reinforced Plastics and Electrical Insulating Materials.

[27] Li F., Zhao C., Deng Z. A Creep Testing Device for Wood Plastic Composites.

[28] (2004). Standard Guide for Evaluating Mechanical and Physical Properties of Wood-Plastic Composite Products.

[29] Du H., Wang W., Wang H., Wang Q. (2015). Influence of wood fiber content on the creep behavior of wood fiber-plastic composite. Jianzhu Cailiao Xuebao/J. Build. Mater..

[30] Li F., Jun Y. (2012). The Reliability Influence of Wood Powder Contenton on Wood-plastic Composite Material. For. Sci. Technol..

